# Imipramine Treatment Alters Sphingomyelin, Cholesterol, and Glycerophospholipid Metabolism in Isolated Macrophage Lysosomes

**DOI:** 10.3390/biom13121732

**Published:** 2023-12-01

**Authors:** Jacob M. Albright, Matthew J. Sydor, Jonathan Shannahan, Christina R. Ferreira, Andrij Holian

**Affiliations:** 1 Department of Biomedical and Pharmaceutical Sciences, Center for Environmental Health Sciences (CEHS), University of Montana, Missoula, MT 59812, USA; 2 Department of Biomedical and Pharmaceutical Sciences, Center for Biomolecular Structure and Dynamics, University of Montana, Missoula, MT 59812, USA; 3 School of Health Sciences, Purdue University, West Lafayette, IN 47907, USA; jshannah@purdue.edu; 4 Metabolite Profiling Facility, Bindley Bioscience Center, Center for Analytical Instrumentation Development, Purdue University, West Lafayette, IN 47907, USA; cferrei@purdue.edu

**Keywords:** lipids, lipidomics, sphingolipids, ceramide, phosphatidylcholine, lysophosphatidylcholine, anisotropy

## Abstract

Lysosomes are degradative organelles that facilitate the removal and recycling of potentially cytotoxic materials and mediate a variety of other cellular processes, such as nutrient sensing, intracellular signaling, and lipid metabolism. Due to these central roles, lysosome dysfunction can lead to deleterious outcomes, including the accumulation of cytotoxic material, inflammation, and cell death. We previously reported that cationic amphiphilic drugs, such as imipramine, alter pH and lipid metabolism within macrophage lysosomes. Therefore, the ability for imipramine to induce changes to the lipid content of isolated macrophage lysosomes was investigated, focusing on sphingomyelin, cholesterol, and glycerophospholipid metabolism as these lipid classes have important roles in inflammation and disease. The lysosomes were isolated from control and imipramine-treated macrophages using density gradient ultracentrifugation, and mass spectrometry was used to measure the changes in their lipid composition. An unsupervised hierarchical cluster analysis revealed a clear differentiation between the imipramine-treated and control lysosomes. There was a significant overall increase in the abundance of specific lipids mostly composed of cholesterol esters, sphingomyelins, and phosphatidylcholines, while lysophosphatidylcholines and ceramides were overall decreased. These results support the conclusion that imipramine’s ability to change the lysosomal pH inhibits multiple pH-sensitive enzymes in macrophage lysosomes.

## 1. Introduction

Macrophages are key regulators of acute and chronic inflammation. Activated macrophages initiate and maintain inflammation by secreting pro-inflammatory cytokines, resulting in chronic inflammation leading to tissue injury. Chronic inflammation is a primary contributor to most human diseases, and the dysfunction of macrophage lysosomes prevents the resolution of inflammation in numerous chronic diseases. For example, amyloid β plaques in Alzheimer’s disease [1], cholesterol crystals in atherosclerosis [2], monosodium urate crystals in gout [3], and crystalline silica particles in silicosis [4] are all associated with macrophage lysosomal dysfunction. Therefore, macrophages are key in multiple chronic inflammatory diseases caused by the uptake of crystalline particles of both endogenous and exogenous origin. However, the mechanisms of macrophage regulation in the inflammatory response are incompletely understood. Further elucidation of how lysosomes function and regulate macrophage-mediated inflammation is needed to inform the development of potential therapeutics. 

Lysosomes are membrane-bound organelles that are responsible for degrading phagocytosed material and have important roles in cellular metabolism, intracellular signaling, and immune responses [5,6]. Over 60 distinct hydrolytic enzymes have been described in lysosomes, which are capable of degrading a wide range of extra- and intracellular material [7,8]. Importantly, the lysosome is the primary organelle for multiple lipid metabolic pathways that have been linked to inflammation and disease. To date, over 70 lysosomal storage disorders have been described and characterized by the overaccumulation of lysosomal lipids caused by genetic deficiencies in lipid catabolizing enzymes [9], many of which are associated with aberrant sphingolipid metabolism, thereby promoting inflammation [10]. The lysosome is also the main organelle for sphingomyelin metabolism in which acid sphingomyelinase (aSMase) cleaves sphingomyelin to produce phosphorylcholine and ceramide. Sphingomyelin and ceramide have been reported to have contrasting roles in regulating inflammation. The inhibition of aSMase or treatment with exogenous sphingomyelin in endotoxin-stimulated macrophages has been shown to block inflammation while exogenous ceramide treatment promotes inflammation in endotoxin-stimulated macrophages [11,12,13]. 

Imipramine is an FDA-approved antidepressant that has been in use since the 1950s [14]. It is representative of a group of cationic and amphiphilic drugs (CADs). CADs, including imipramine, can freely diffuse across cellular membranes and, once protonated inside the acidic lysosome lumen, become trapped and accumulate, thereby increasing the lysosomal pH [15]. As an inhibitor of aSMase, imipramine is expected to increase lysosomal sphingomyelin and decrease ceramide levels. Previous work by our laboratory indicated that imipramine treatment in bone marrow-derived macrophages increased lysosomal cholesterol levels, which decreased crystalline silica-induced lysosome membrane permeabilization (LMP), subsequent inflammation, and cell death [16]. It was concluded that the imipramine-induced increase in lysosomal cholesterol stabilized the lysosome membrane, preventing LMP and subsequent inflammation. This conclusion was supported by multiple studies, demonstrating that increases in lysosomal membrane cholesterol induced biophysical membrane changes imparting resistance to LMP [17,18,19]. Further support came from an *in vivo* study in which imipramine pre- and post-treatment blocked crystalline silica-induced inflammation and lung pathology in C57BL/6 mice [20]. Therefore, inhibiting lysosomal aSMase with imipramine has the potential to influence not only sphingomyelin metabolism, but also the cholesterol content, inducing changes to the lysosome membrane that are protective against LMP. However, the effects of imipramine or any other CAD on lipid metabolism have not yet been demonstrated in isolated macrophage lysosomes. 

Therefore, this study investigated the hypothesis that, in isolated macrophage lysosomes, imipramine would disrupt sphingomyelin metabolism by inhibiting aSMase, increasing sphingomyelin and cholesterol while decreasing ceramide. Ultracentrifugation was used to isolate lysosomes from alveolar macrophages that were expanded *ex vivo* (mexAM) and pretreated with imipramine. An in-depth assessment of lysosome purity, integrity, and functionality was conducted to validate the lysosome isolation procedure. Subsequently, mass spectrometry was used to assess the lipid composition of the isolated lysosomes. In addition, time-resolved fluorescence anisotropy was used to measure changes in the lipid order of the isolated lysosomes following the lysosomal cholesterol accumulation induced by treatment with the drug U18666A. 

## 2. Materials and Methods

### 2.1. Cell Culture

The murine *ex vivo* cultured alveolar macrophages (mexAMs) were generated according to the Gorki et al. protocol [21]. Briefly, primary AMs were isolated via whole lung lavage from male and female 6–8-week-old C57BL/6 mice and pooled. The pooled cells were cultured in an RPMI complete media with 10% FBS and 1% Antibiotic-Antimycotic (cat # 15240096, penicillin/streptomycin/Amphotericin B, Thermo Fisher Scientific, Waltham, MA, USA) for a final concentration of 100 units/mL of penicillin, 100 μg/mL of streptomycin, and 250 ng/mL of Gibco Amphotericin B at 37 °C in 5% CO_2_ with the addition of the following growth factors: murine GM-CSF (30 ng/mL, cat # 250-05, Peprotech, Cranbury, NJ, USA), recombinant human TGFβ (10 ng/mL, cat # 100-36E, Peprotech), and rosiglitazone (1 µM, cat # 122320-73-4, Sigma, St. Louis, MO, USA). Once an 80% confluency was reached, the cells were reseeded with fresh growth factors until the required number of cells was obtained for the lysosome isolations, 50 × 10^6^ cells/sample. To aid in the detachment from the flask bottom, the mexAMs were incubated with a local anesthetic lidocaine (20 mM, cat # J63035.14, Thermo Fisher Scientific) for 4 min at 37 °C and then gently scraped from the flask bottom. The usage of lidocaine for the detachment of mexAMs and other cell types has been used previously by our laboratory and others and has been shown to preserve the cell surface proteins that are otherwise lost through trypsinization or scraping alone [21,22,23]. For the drug treatments, the mexAMs were incubated at a cell density of 10^6^ cells/mL in a complete media without growth factors, with either imipramine (25 µM, cat # 113-52-0, Cayman Chemical, Ann Arbor, MI, USA) or U18666A (1 µg/mL, cat # 3039-71-2, Cayman Chemical) for 24 h prior to the lysosome isolations. 

### 2.2. Lysosome Isolation

The purification of the lysosomes was accomplished via density gradient ultracentrifugation utilizing a lysosome isolation kit (cat # 89839, Thermo Fisher Scientific). Briefly, 50 × 10^6^ cells (mexAM)/sample were collected using the same procedure outlined above and pelleted by centrifuging for 5 min at 500× *g*. Using primary AM would have been prohibitive for obtaining the high cell numbers needed to isolate the lysosomes. The cell pellets were resuspended in 800 µL of lysosome enrichment reagent A (included in kit) and vortexed for 5 s before incubating on ice for exactly 2 min. A glass Dounce homogenizer with a tight-fitting type B pestle was used to mechanically lyse the cells by performing 30, 50, or 70 strokes on the ice. The cell lysates were transferred to 800 µL of ice-cold lysosome enrichment reagent B (included in kit) and then centrifuged for 10 min at 500× *g* in 4 °C. The supernatants were carefully overlayed atop 8.5 mL of a 17% OptiPrep cell separation media (included in kit) in 10.4-mL ultracentrifuge tubes. A Beckman Coulter ultracentrifuge (model: Optima XE-90) with a type Ti 70.1 rotor was used and centrifuged at 145,000× *g* for 1 h at 4 °C. The lysosome rich bands were collected in their entirety by carefully aspirating with a pipettor and then diluted by a factor of three with a synthetic cytosol buffer made to simulate the intracellular ion environment (140 mM of KCl, 20 mM of NaCl, 2 mM of MgCl, 1 mM of EGTA, 10 mM of Tris, and a pH of 7.35). The lysosome pellets were formed by centrifuging the diluted fractions at 14,000× *g* for 30 min at 4 °C, washed once with a cytosol buffer, and resuspended in 100 uL of the cytosol buffer. The lysosomes suspended in the cytosol buffer were frozen at −80 °C until use in the following experiments. 

### 2.3. Western Blot

**Figure 1:**
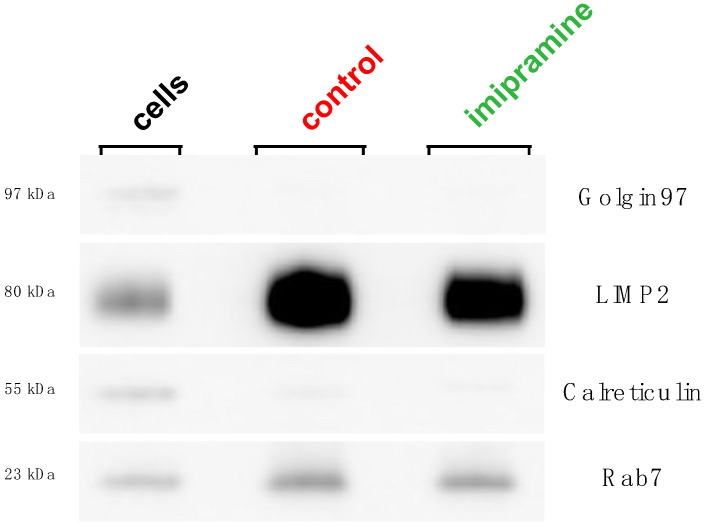
Western blot analysis of lysosome purity. The lysosomes were isolated from the control and imipramine-treated mexAMs using density gradient ultracentrifugation and compared to the cell lysate (cells). Protein of equal weight (10 µg) was loaded into each lane. The bands represent the organelle-specific antibodies: lysosomes (LIMP2), ER (Calreticulin), Golgi, (Golgin97), and late endosomes (Rab7). Two PVDF membranes were imaged simultaneously under the same conditions to produce a single image (Figure S3).

Western blot was used to verify the purity of the lysosome fractions. The isolated lysosomes or cell lysates were lysed with a RIPA assay buffer (20 mM, cat # 89901, Tris-Cl, 150 mM NaCl, 1% Nonidet P-40, 0.5% sodium deoxycholate, 0.1% SDS) plus Halt protease inhibitor cocktail (cat # 87785, Thermo Fisher Scientific). The total protein concentration was determined using the Pierce BCA protein assay kit (cat # 23227, Thermo Fisher Scientific). Equal amounts of the protein (10 µg/well) were run on NuPAGE 4–12% Bis-Tris mini protein gels (cat # NP0322, Invitrogen, Thermo Fisher Scientific) in a NuPAGE MOPS SDS running buffer (cat # NP0001, Thermo Fisher Scientific) at 200 V for 50 min, followed by a traditional wet transfer onto a polyvinylidene difluoride (PVDF) membrane at 100 V for 1 h. The PVDF membranes were blocked with 5% non-fat dry milk in TBST for 1 h and then incubated with the primary antibody overnight at 4 °C. ECL DualVue Western blotting markers (cat # RPN810, Cytiva, Marlborough, MA, USA) were used for the signal detection. The primary antibodies were used to detect specific organelle markers as follows: lysosome (LIMP2/SCARB2 Rabbit mAb, cat # 27960, Cell Signaling, Danvers, MA, USA), ER (Calreticulin Rabbit Ab, cat # 2891, Cell Signaling), Golgi (Golgin-97 Rabbit mAb, cat # 13192, Cell Signaling), and late endosome (Rab7 Rabbit mAb, cat # 9367, Cell Signaling). The secondary antibody used was donkey anti-rabbit IgG HRP (cat # 406401, Biolegend, San Diego, CA, USA). For all the western blot images, two PVDF membranes were exposed simultaneously to produce a single image using an ImageQuant LAS biomolecular imager set to the sensitive setting. As shown in Figure 1, the brightness/contrast maximum was decreased from 65,535 to 38,943 using the ImageJ software version 1.53t to ensure complete band detection was accomplished, and to remove obvious pixelation from the LIMP2 protein bands, as seen in the original image. This adjustment did not obscure or eliminate any information from the original image, which can be found along with the brightness-adjusted image in the supplemental section (Figure S3a,b).

### 2.4. LysoTracker Red and NAG Enzyme Activity 

LysoTracker Red DND-99 (LTR) (cat # L7528, Thermo Fisher Scientific) and the N-acetyl-β-d-glucosaminidase (NAG) enzyme activity were used to validate the lysosome integrity and functionality. The enzyme NAG has been used previously in multiple studies as a marker for lysosome membrane permeabilization [16,18,24]. In this study, the NAG activity in the control lysosomes relative to the fully lysed lysosomes was used as an indicator of the lysosome integrity. Furthermore, NAG is a relatively large lysosomal enzyme (~140 kDa), suggesting that if it had leaked from lysosomes, other smaller enzymes such as cathepsin D (~43 kDa) will have also leaked. The lysosome samples were diluted by a factor of 10 in a cytosol buffer and incubated with 100 nM of LTR for 30 min in the dark at 22 °C. The fluorescence was detected in a Biotek Cytation 5 microplate fluorimeter from Agilent Technologies (Santa Clara, CA, USA) with an excitation of 561 nm and an emission of 602 nm, both of which were a 20 nm bandwidth. All the samples were plated in duplicate or triplicate. The activity of the lysosomal enzyme NAG was measured in the unperturbed lysosome samples or lysosomes lysed with the detergent Triton-x100. To measure the NAG activity, the lysosome samples were diluted by a factor of 10 and incubated for 30 min in the dark at 22 °C with or without 0.1% Triton-x100 and 0.8 mM of the NAG substrate 4-Methylumbelliferyl-2-acetamido-2-deoxy-β-D-Glucopyranoside (cat # 26953, Cayman Chemical) in 0.2 M citrate. The fluorescence was detected in a SpectraMax iD3 microplate fluorimeter from Molecular Devices (San Jose, CA, USA) with an excitation of 356 nm and an emission of 444 nm. All the samples were plated in duplicate. 

### 2.5. Cryo-Electron Microscopy

The samples were plunge frozen on Quantifoil 200 mesh R2/1 Cu grids using a Vitrobot Mark IV plunge freezer set at 4 °C and 95% humidity with cellulose blotting paper on both sides. The sample sizes were 4 µL and the blot times were 4 s at blot force 5. The frozen grids were then imaged on a Talos Arctica transmission electron microscope operating at 200 kV. The images were recorded using a Gatan K3 camera at a magnification of 21,000× and a defocus value of −3.0 µm. The exposure times were 3.5 s at approximately 15 electrons per pixel per second. The magnified pixel size was 3.033 e-/Å2 and the total dose was 45 e-/Å2.

### 2.6. Lipid Extraction

The lipids were extracted from the isolated lysosomes via the Bligh–Dyer lipid extraction method [25]. Briefly, methanol and chloroform were added to 200 µL of each lysosome sample. The mixtures were vortexed and incubated at 4 °C for 15 min prior to the addition of purified water and additional chloroform. The solution was then centrifuged at 5000× *g* for 10 min, resulting in the separation of the solution into two phases: an aqueous alcoholic top phase and an organic chloroform bottom phase. The bottom phase was isolated and concentrated to dryness using a Speedvac, producing the dried lipid extracts. 

### 2.7. Time-Resolved Fluorescence Anisotropy Measurements

The isolated lysosomes were diluted by a factor of 10 in a synthetic cytosol buffer. Di-4-ANEPPDHQ (cat # D36802, Thermo Fisher Scientific) was used as the fluorescence probe and was added to the isolated lysosomes for a final concentration of 400 nM. The lysosomes and Di-4-ANEPPDHQ were incubated at 37 °C +/−0.02 °C for 15 min to ensure the incorporation of the probe into the membrane. The anisotropy measurements were acquired using a custom-built Quantum Northwest (Liberty Lake, WA, USA) time-resolved fluorimeter, as previously described [26]. A 470 nm pulsed diode laser (PicoQuant LDH-P-C-470) with a repetition rate of 5 MHz was used for the excitation of Di-4-ANEPPDHQ. The fluorescence emissions were collected using a 500 nm longpass filter (Chroma, Bellows Falls, VT, USA) and an Edinburgh Instruments (Livingston, UK) photomultiplier tube (H10720-01). All the data was fitted as an anisotropy reconvolution using the PicoQuant (Berlin, Germany) FluoFit 4.6.6 software. The wobble-in-a-cone angle was previously defined and was calculated by the following equation [27].

$$S^{2}=\frac{r_{\infty }}{r_{0}}={\left[\frac{1}{2}\cos\left(\theta \right)\left(1+\cos\left(\theta \right)\right)\right]}^{2}$$

The order parameter *S* is calculated by the ratio of the residual anisotropy (*r*_∞_) and the initial anisotropy (*r*_0_). This ratio also allows for the calculation of the wobble-in-a-cone angle (*θ*). 

### 2.8. Data Acquisition and Statistical Analysis 

#### 2.8.1. LysoTracker Red, NAG Enzyme Activity, and Time-Resolved Fluorescence Anisotropy Data

The statistical significance was defined by the probability of type I error occurring at less than 5% (*p* < 0.05). An unpaired student t-test was used for the comparison of two means in all instances. Three technical replicates were averaged for each n. Graphics and statistical analyses were performed on the PRISM version 10 software (GraphPad, San Diego, CA, USA). 

#### 2.8.2. Lipid Profiling 

The lipid extracts were resuspended in 200 µL of an injection solvent consisting of a 3:6.65:0.35 mixture of acetonitrile:methanol:ammonium acetate. The samples were then diluted by a factor of 50 with an injection solvent. A volume of 10 µL of the diluted sample was used for the flow injection using a micro-autosampler (G1377A) to the ESI source of an Agilent 6410 triple quadrupole mass spectrometer (Agilent Technologies, Santa Clara, CA, USA). A capillary pump was connected to the autosampler and operated at a flow rate of 10 µL/min and a pressure of 150 bar. The capillary voltage on the instrument was 5kV and the gas flow was 5.1 L/min at 300 °C. The multiple reaction monitoring (MRM) profiling approach was utilized to evaluate the lipid components of the macrophage lysosomes using the recently reported methods [28,29]. The Equisplash Lipidomics Internal Standard, Avanti p/n 330731 (1 ng of each lipid in the mix), was used as a quality control sample to monitor the instrument performance. Pure methanol was used to flush the system between the injection of the different samples. The MRM profiling approach was based on using class diagnostic fragments to detect the lipids of diverse classes listed in the Lipid Maps database by flow injection. The lipids were detected at the species level structure and their profile in the samples was used for the statistical analysis [30,31,32,33,34]. This method was a shotgun lipidomics approach for lipid profiling, which has been validated using LC-MS/MS [34,35]. The use of a list of MRM scans or ion transitions instead of a full mass scan caused less matrix interference than normally observed for targeted vs. full scan mass analyses since only the selected ions and fragments were transmitted to the detector. The MS data obtained were processed using an in-house script to generate a list of MRM transitions with their respective sum of absolute ion intensities over the acquisition time. Synthetic cytosol (lysosome isolation media) was used as the background samples for the assessment. Only the lipids present in all three samples from a group with ion intensities that were at least three times higher than the synthetic cytosol for the alveolar macrophage samples were included in the analysis. The data were analyzed and normalized by the ion signal of the internal standard. A statistical analysis was performed utilizing Metaboanalyst 5.0 (http://www.metaboanalyst.ca/, accessed on 30 March 2023). The relative amounts were log auto-scaled to obtain a normal distribution, and evaluated by univariate analysis, principal component analysis (PCA), and cluster analysis/heatmap. The relative abundance of the identified lipids for each sample was determined by comparing them to the average of the control macrophage lysosomes. The comparisons of the lipid abundance differences between the IMP-treated and control macrophage lysosomes were determined using a student’s t-test; *p* < 0.05. 

## 3. Results

### 3.1. Validation of Lysosome Purity

Several lysosome isolation protocols have been described, each with their own benefits and limitations. Density gradient ultracentrifugation is a well-established technique that does not involve the addition of foreign material or the alteration of the lysosome’s native protein or lipid composition. To determine the changes in the lysosome lipid composition induced by imipramine, the lysosomes were isolated from murine *ex vivo* alveolar macrophages (mexAM) using density gradient ultracentrifugation. Isolating intact lysosomes requires the lysis of the plasma membrane with minimal damage to the lysosomes. Thus, in a controlled fashion, the plasma membranes were mechanically lysed using a Dounce homogenizer. The optimal number of Dounce strokes to obtain the highest lysosome purity was assessed using a western blot analysis. First, the ratio of the lysosome-specific protein LIMP2 to the ER-specific protein Calreticulin was determined in the isolated lysosomes and cell lysates. Then, the LIMP2/Calreticulin ratio in the lysosomes was divided by the LIMP2/Calreticulin ratio in the cell lysates to calculate the fold purity. This was done using three different Dounce stroke numbers (30, 50, and 70). The optimal number of Dounce strokes was determined to be 30 strokes, yielding the highest fold purity of approximately 20-fold (Figure S1 and Table S1). Therefore, 30 strokes were used to lyse control and imipramine-treated cells for the rest of the study. Next, we assessed whether imipramine treatment had an impact on lysosome purity. There was no obvious change in the band sedimentation of lysosomes due to imipramine treatment (Figure S2). While there was a small visible decrease in the LIMP2 protein in the imipramine samples relative to the control (Figure 1), a decrease of similar magnitude was detected in the Calreticulin protein when all the protein bands were quantified using ImageJ software version 1.53t (Table S2). The protein quantitation resulted in a slightly higher LIMP2/Calreticulin ratio in the lysosomes from imipramine-treated cells relative to lysosomes from control cells. However, the LIMP2/Calreticulin ratios were not significantly different in the imipramine lysosomes compared to the control lysosomes, suggesting there was no change in lysosome purity caused by the imipramine treatment. 

### 3.2. Validation of Lysosome Integrity, Functionality, and Morphology

**Figure 2:**
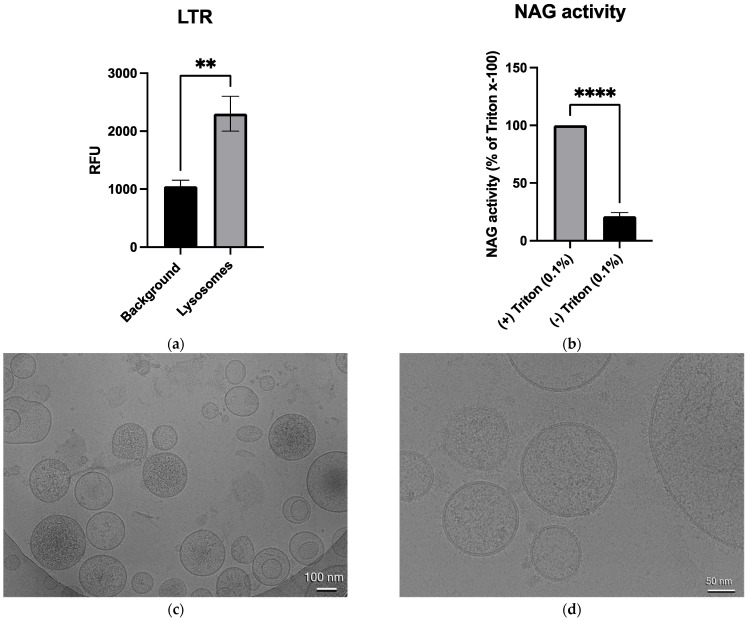
The lysosome integrity, functionality, and morphology were assessed. The isolated lysosomes had a significant approximately two-fold increase over the background LysoTracker Red (LTR) fluorescence (** indicates *p* < 0.01), n = 5 (**a**). The N-acetyl-β-d-glucosaminidase (NAG) activity was significantly increased in the isolated lysosomes that underwent complete lysis via incubation with 0.1% of Triton x-100 for 30 min (**** indicates *p* < 0.0001), n = 3 (**b**). Student t-tests were used to determine the statistical significance (mean ± *SEM* for all the data). The representative cryo-electron microscopy images of the lysosomes isolated from the control mexAMs indicated the majority of the lysosomes were spherical in morphology and intact with no apparent contamination from non-lysosome organelles (**c**). Distinct lipid bilayers were visible (**d**).

LysoTracker Red (LTR) fluorescence intensity and N-acetyl-β-d-glucosaminidase (NAG) enzyme activity were used to determine lysosome integrity and functionality since the lysosome membrane must remain intact to accumulate LTR and functioning lysosomes require active hydrolytic enzymes, such as NAG [36,37]. The fluorescence intensity of LTR was measured in the control lysosomes, resulting in a significant approximately two-fold increase over the background fluorescence (Figure 2a). The enzymatic activity of NAG was determined by measuring the fluorescence intensity of the cleaved NAG substrate 4-Methylumbelliferyl-2-acetamido-2-deoxy-β-D-Glucopyranoside. In the control lysosomes, there was a significant decrease in the NAG activity relative to the lysosomes that underwent total lysis via treatment with the detergent Triton x-100 (0.1%). The absence of NAG enzyme activity in the control lysosomes indicated that the majority of lysosomes maintained their integrity and functionality until they were purposely lysed (Figure 2b). Lysosome morphology was assessed using cryo-electron microscopy (Cryo-EM). As seen in the Cryo-EM images, the lysosomes maintained a mostly spherical structure with no apparent contamination of non-lysosome organelles (Figure 2c). Furthermore, distinct lipid bilayers were intact, which further supported the notion that the lysosomes maintained their integrity (Figure 2d). Taken together, these results suggested that the lysosomes used in this study were of a high purity, with minimal perturbance due to imipramine treatment, and maintained structural integrity and functionality. Therefore, the lysosomes were appropriate models for measuring the effects of imipramine on lysosome lipid metabolism. 

### 3.3. Imipramine Treatment Altered Sphingomyelin, Cholesterol, and Glycerophospholipid Metabolism in Macrophage Lysosomes

#### 3.3.1. Principal Component Analysis and Heatmap

**Figure 3:**
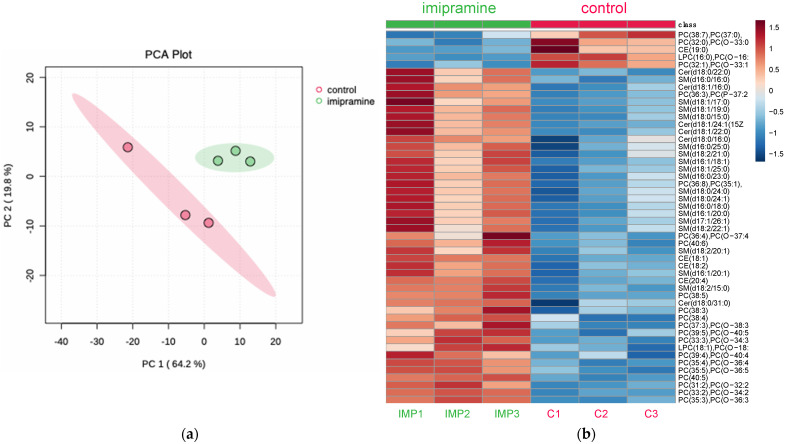
Principal component analysis (PCA) and heatmap generated from the lipid profiles of the isolated lysosome samples. The lysosomes were isolated from the mexAMs following the 24-h imipramine treatment (25 μM). The PCA revealed a tight grouping of the imipramine-treated (green) and control lysosomes (red), (**a**). Imipramine caused changes to the lipid profile of the isolated macrophage lysosomes with the majority of changes representing increases in the differential expression (**b**), (*p* < 0.05), n = 3.

To determine the ability of imipramine to induce changes to lysosome lipid metabolism, the mexAMs were incubated with imipramine (25 µM) for 24 h prior to lysosome isolation and lipid analysis. Mass spectrometry was performed on the imipramine-treated and control lysosomes using multiple reaction monitoring (MRM) profiling to measure the overall changes in cholesterol esters, sphingomyelins, ceramides, phosphatidylcholines, and lysophosphatidylcholines. Principal component analysis depicted a clear differentiation between the imipramine-treated and control lysosomes (Figure 3a). The heatmap analysis was used to determine the overall changes to the lysosome lipid profile. In summary, 4 cholesterol esters, 12 sphingomyelins, 2 ceramides, 25 phosphatidylcholines (PCs), and 2 lysophosphatidylcholines (LPCs) at the species level structure were differentially expressed (*p* < 0.05) (Figure 3b). These results indicated that imipramine induced significant changes to sphingomyelin, cholesterol, and glycerophospholipid metabolism in the macrophage lysosomes.

#### 3.3.2. Lipid Species Fold-Changes

**Figure 4:**
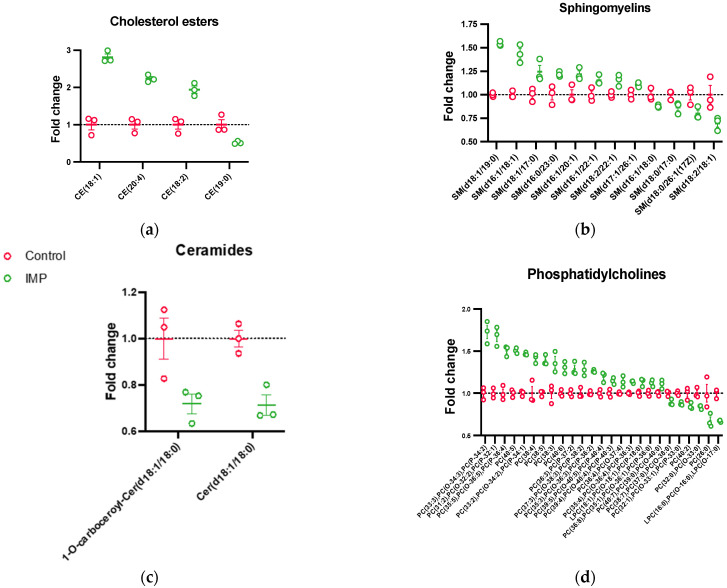
Imipramine-induced differential expression of the lipid species. The data are presented as imipramine-induced fold-changes relative to the lysosomes that were isolated from the control mexAMs (Table S3). The cholesterol esters (CE), (**a**); sphingomyelins (SM), (**b**); ceramides (Cer), (**c**); phosphatidylcholines (PC & LPC), (**d**) were differentially expressed as a result of the 24-hr imipramine (25 μM) treatment. Student t-tests were performed to determine the statistical significance (mean ± *SEM*, *p* < 0.05), n = 3.

To identify potential trends in the differentially expressed lipids caused by imipramine treatment, lipids were grouped by class and species fold-changes were plotted (Figure 4). Sphingomyelins, cholesterol esters, and ceramides were analyzed due to their ability to modulate membrane fluidity and inflammatory pathways [12,16,38]. Imipramine treatment increased the cholesterol esters CE (18:1), CE (20:4), and CE (18:2), accounting for the highest fold-changes observed (Figure 4a). Since imipramine is a functional inhibitor of aSMase, an increase in sphingomyelin and a decrease in ceramide was hypothesized. Of the 12 sphingomyelin and 2 ceramide species that were differentially expressed, 8 sphingomyelins were increased (Figure 4b) and both ceramides decreased (Figure 4c). These results corroborated previous work by our laboratory, which suggested that imipramine treatment increased the lysosomal cholesterol content [16] while providing evidence to support that imipramine inhibited lysosomal aSMase. PC has been reported to be the most abundant lipid in cellular membranes, playing numerous roles in health and disease, including being a precursor for highly pro-inflammatory lipids such as arachidonic acid and LPC [39,40,41,42]. Interestingly, in this study, PCs had the highest number of altered lipid species, with the majority elevated relative to the control (Figure 4d). Of the PCs with an increased expression, approximately 90% were unsaturated, while approximately 80% of the downregulated PCs were fully saturated. In addition, 2 LPCs were differentially expressed, which could have important implications in imipramine’s biological effects (Figure 4d). Altogether, these results suggested that imipramine extensively altered lipid metabolism in the macrophage lysosomes, which could induce biophysical changes to the lysosome membrane effecting membrane function. To our knowledge, this was the first study to measure the broad effects of imipramine treatment on the lipid profile of isolated macrophage lysosomes. 

### 3.4. Cholesterol Accumulation Induced Biophysical Changes in Isolated Macrophage Lysosomes

Previous studies have shown that increasing lysosomal cholesterol content stabilized the lysosome membrane, preventing particle-induced LMP and downstream inflammation [16,18]. Moreover, changes in the lipid content of specific membrane lipids (e.g., cholesterol, sphingomyelin, and phosphatidylcholine) in model membrane systems induced biophysical changes, such as alterations in the lipid order [38,43,44]. Since the lipid composition of the lysosomes from the imipramine-treated cells was altered from the control lysosomes, time-resolved fluorescence anisotropy was used to determine whether the changes would result in measurable changes to the lipid order in the isolated lysosomes, which could help define a mechanism of resistance against particle-induced LMP. The wobble-in-a-cone angle of the solvatochromic and lipophilic fluorescence probe Di-4-ANEPPDHQ was used to assess the lipid order of the isolated lysosomal membranes. The isolated lysosomes were incubated with Di-4-ANEPPDHQ at a concentration of 400 nM prior to the time-resolved fluorescence anisotropy measurements. The wobble-in-a-cone angle of the lysosomes isolated from the control and imipramine-treated cells was measured and showed no significant difference between the means. However, many lipids were altered after the imipramine treatment, which could have opposing roles in the lipid order and result in an overall similar lipid order to the control lysosomes as Di-4-ANEPPDHQ partitions into both the ordered (L_o_) and disordered (L_d_) phases of the membrane [44]. After detecting increased cholesterol esters as a result of the imipramine treatment, the U18666A treatment of the mexAMs was used as a targeted approach to increase cholesterol more selectively in order to demonstrate that CAD treatment can change the lipid order in lysosomes. The CAD U18666A is an inhibitor of the NPC1 cholesterol transport protein and has been reported to increase lysosomal cholesterol [17,18,45]. The results of the time-resolved anisotropy measurements of the lysosomes isolated from the U18666A-treated cells showed a significantly decreased wobble-in-a-cone angle (*p* value: 0.02) of 27.76 ± 0.378 degrees relative to 31.02 ± 1.47 degrees measured in the control lysosomes. This result was consistent with an increase in lysosomal cholesterol, causing an increase in the lipid order. 

## 4. Discussion 

Macrophages are central cells present in various tissues that are involved in the regulation of inflammatory responses [1,2,3,4]. The mexAM cell model used in this study represented primary AMs phenotypically, transcriptionally, and morphologically [21,22]. Furthermore, when engrafted into AM-depleted mice, the mexAMs were able to restore lung function in an alveolar proteinosis disease model, illustrating the in vivo translatability of these AMs [21]. Macrophage inflammation is regulated by lysosome integrity and, consequently, the sphingomyelin-ceramide-sphingosine-1-phosphate pathway, which is critical in macrophage responses to particle-induced inflammation [20,46]. Previous studies demonstrated that CADs accumulate in lysosomes, disrupt normal SM metabolism, and elevate cholesterol in lysosomes [16,47]. Furthermore, CADs have been reported to block LMP induced by crystalline silica particles [16,18]. However, since CADs are not specific sphingomyelin pathway inhibitors, more lysosomal-specific information is necessary to better understand the CAD mechanisms of action, impacts on lysosomal lipid metabolism, and effects on lysosomal function and LMP. A complication in any study of sphingolipid metabolism in lysosomes was that this pathway is not unique to lysosomes. Therefore, the purpose of this study was to determine the effects of the CAD imipramine on sphingomyelin metabolism in isolated lysosomes and evaluate the potential changes in membrane function through changes in the lipid order. To achieve this, a lysosome preparation from mexAMs was first developed and evaluated. Second, mass spectrometry was used to measure the changes in the lipid content of macrophage lysosomes following the imipramine treatment. The results demonstrated that imipramine significantly increased the ratios of cholesterol esters, sphingomyelins, and PCs, and overall decreased ceramides and LPCs. Finally, the increased cholesterol content in the lysosomes was shown to impact the lysosomal membrane properties and, likely, membrane function.

The current findings provide evidence for the ability of imipramine to inhibit aSMase, preventing the catabolism of sphingomyelin and the generation of ceramide in lysosomes while also increasing the lysosomal cholesterol content. The increase in cholesterol esters was postulated because the previous work reported that imipramine blocked lysosome-mediated inflammation and that imipramine treatment induced free cholesterol accumulation in lysosomes [16]. Furthermore, it was demonstrated that the accumulation of free cholesterol resulted in a concomitant increase in cholesterol esters in lysosomes [48]. Therefore, free cholesterol and cholesterol esters can be coregulated in lysosomes. In addition, cholesterol ester accumulation was reported in the lysosomes isolated from the Niemann Pick protein 1 knockout cells [49]. Niemann–Pick type C1 and C2 proteins (NPC1/2) work in conjunction to facilitate cholesterol export from the lysosome [50], and aSMase inhibition has been shown to block the function of NPC2 [51], suggesting that the aSMase activity is connected to free cholesterol export from the lysosome. Therefore, in the present study, it was possible that imipramine inhibited aSMase, resulting in NPC2 inhibition, and thus causing the accumulation of free cholesterol and, consequently, cholesterol esters. In addition, since imipramine is a CAD and increases the lysosomal pH [15], an alternate explanation for the observed increase in cholesterol esters was that the imipramine-induced pH changes indirectly inhibited the catalytic activity of lysosomal acid lipase, the enzyme responsible for cleaving cholesterol esters from low density lipoproteins in macrophage lysosomes [52]. Therefore, it was likely that imipramine increased the lysosomal cholesterol levels by disrupting both lysosome lipid metabolism and lipid transport. Future studies will be needed to elucidate the mechanism of imipramine-induced lysosomal cholesterol accumulation with respect to the role of specific cholesterol transport proteins, such as NPC1 and NPC2, and the possibility of imipramine inhibiting lysosomal acid lipase by decreasing lysosomal acidity. 

As mentioned above, several lipid classes were significantly altered by the imipramine treatment, including ceramide, PC, and LPC. The decrease in ceramides was predicted since blocking the aSMase activity would be expected to decrease ceramide production, a product of aSMase. However, the changes in PC and LPC were not as easily explained. It was possible that imipramine’s impact on the lysosomal pH affected the activity of multiple acid hydrolases, which by definition required an acidic pH for optimal activity. For example, lysosomal phospholipase A2 (lysoPLA2), which is highly expressed in alveolar macrophages [53], has been shown to function optimally in isolated lysosomes at a pH of ~4.5 with minimal activity at pH > 5.5 [54]. In addition, the CAD amiodarone also increased the lysosomal pH [15], and has been shown to inhibit the lysoPLA2 activity [55]. Thus, it was likely that the imipramine treatment not only inhibited the aSMase enzyme activity but also impacted the activity of other pH-sensitive lysosomal enzymes, such as lysoPLA2, which would explain the observed changes in PC and LPC.

Since the imipramine treatment resulted in changes to the lipid profile of the lysosomes isolated from the mexAMs, the impact of imipramine on the lipid order of the lysosome membranes was assessed. Time-resolved fluorescence anisotropy was used to measure the lipid order via the wobble-in-a-cone angle of Di-4-ANEPPDHQ in the isolated lysosomal membranes. This parameter was reported to be sensitive to changes in the lipid content and order [56,57]. However, there was no significant change to the cone angle of the lysosomes isolated from the imipramine-treated cells in comparison to the lysosomes isolated from the control cells. A possible explanation could be that the large number of lipids that were significantly altered upon imipramine treatment did not significantly change the overall lipid order of the lysosome to an amount detectable by the anisotropy measurements of Di-4-ANEPPDHQ, as this probe partitioned into both the ordered (L_o_) and disordered (L_d_) phases [44]. Since cholesterol esters and likely free cholesterol were both increased with the imipramine treatment, the question remained whether significantly increasing lysosomal cholesterol could result in lipid order changes in lysosomes. Therefore, treatment with the drug U18666A, which was predicted to have a similar but more potent effect on increasing lysosomal cholesterol, was evaluated. The drug U18666A has been reported to increase the lysosomal cholesterol levels by directly inhibiting the NPC1 protein [45]. Supporting the predicted outcome, the U18666A treatment of the lysosomes significantly lowered the cone angle of Di-4-ANEPPDHQ, indicating an increase in the lipid order. This increase in the lipid order following the U18666A treatment was congruent with increased lysosomal cholesterol, which has been previously described [17,18,45]. Other studies using model membranes and cells have demonstrated instances where elevated membrane cholesterol increased the lipid order [43,58,59,60]. Consequently, increasing lysosomal cholesterol can directly affect the lysosome membrane properties, as measured by changes in the lipid order. It is likely that the imipramine treatment also caused similar membrane changes, although they were possibly too subtle and/or localized in different portions of the lysosomes to detect using Di-4-ANEPPDHQ in this model. 

## 5. Conclusions 

In conclusion, these results provide significant new information regarding the role of imipramine on lysosome lipid metabolism in macrophages and the biophysical outcomes of lysosomal cholesterol accumulation. In order to accurately measure the imipramine-induced changes to lysosome lipid metabolism, a procedure was developed to isolate macrophage lysosomes and was validated using multiple techniques. It was demonstrated that imipramine has broad impacts on lysosome lipid metabolism, including alterations in cholesterol, sphingomyelin, and glycerophospholipid metabolism, which have important implications in a range of macrophage-mediated inflammatory diseases associated with the dysregulation of lysosome lipid metabolism and lysosome dysfunction. The increase in the lipid order (decrease in the cone angle) of the isolated macrophage lysosomes caused by cholesterol accumulation could play a role in imipramine’s preventative action against particle-induced inflammation. Future work will be needed to confirm the effects of imipramine-induced changes to lysosome lipid metabolism on macrophage-mediated inflammation. 

## Data Availability

All the data presented in this study are available in the article and supplementary section.

## Supplementary Materials

The following supporting information can be downloaded at: https://www.mdpi.com/article/10.3390/biom13121732/s1. Figure S1: Western blot for the optimization of cell homogenization; Figure S2: Photograph illustrating the similarity in the band sedimentation of the lysosomes isolated from the control and imipramine-treated mexAMs; Figure S3: Original Western blot image and brightness-adjusted image corresponding to Figure 1; Figure S4: Representative VV and VH fits for time-resolved anisotropy measurements of Di-4ANEPPDHQ in the isolated lysosomes; Table S1: Quantitation of the optimal number of Dounce strokes based on the mean gray values of the Western blot protein bands; Table S2: Quantitation of the Western blot image corresponding to Figure 1; Table S3: Lipid species fold-change data corresponding to Figure 4; Table S4: Absolute intensities of lipid species corresponding to Figure 4.
