# Supplementary material for: Imipramine Treatment Alters Sphingomyelin, Cholesterol, and Glycerophospholipid Metabolism in Isolated Macrophage Lysosomes
Source: Biomolecules. 2023 Dec 1;13(12):1732. doi: 10.3390/biom13121732 (PMC10742328; doi:10.3390/biom13121732)
Supplement: Supplementary file 1 [file biomolecules-13-01732-s001.zip › Figure S1.pdf]

Figure S1. Western blot for optimization of cell homogenization. The optimal number of Dounce strokes needed to mechanically lyse mexAM plasma membranes and obtain the highest purity based on relative gray values of LIMP2 and Calreticulin protein bands (a). Original western blot image, labeled (b).

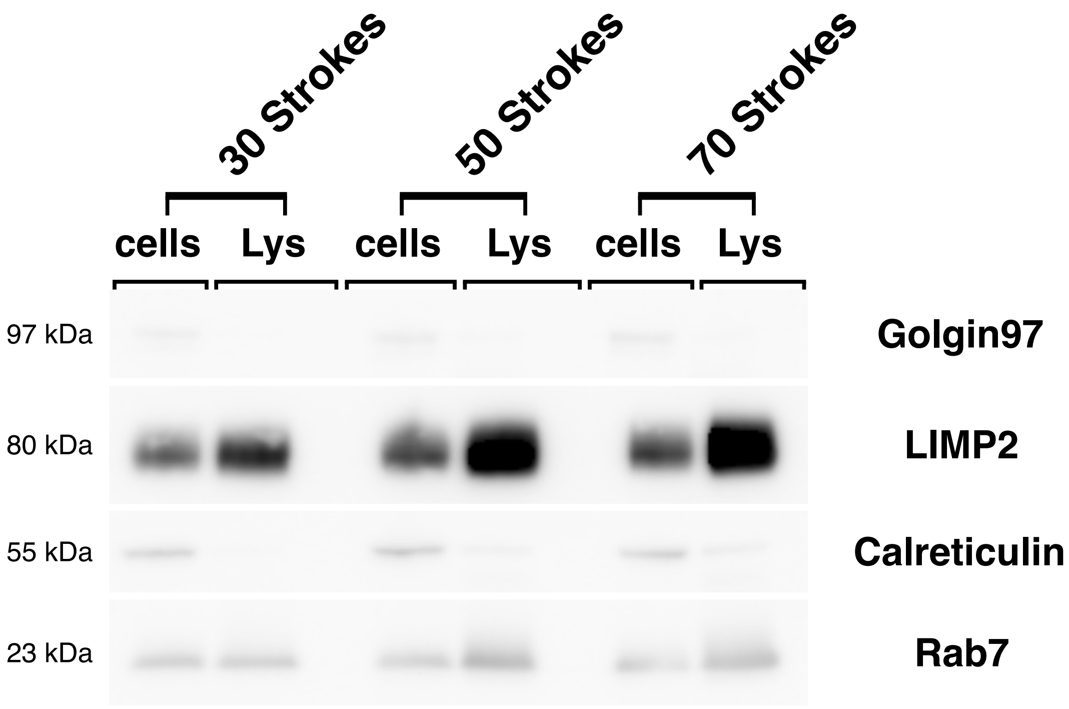

(a)

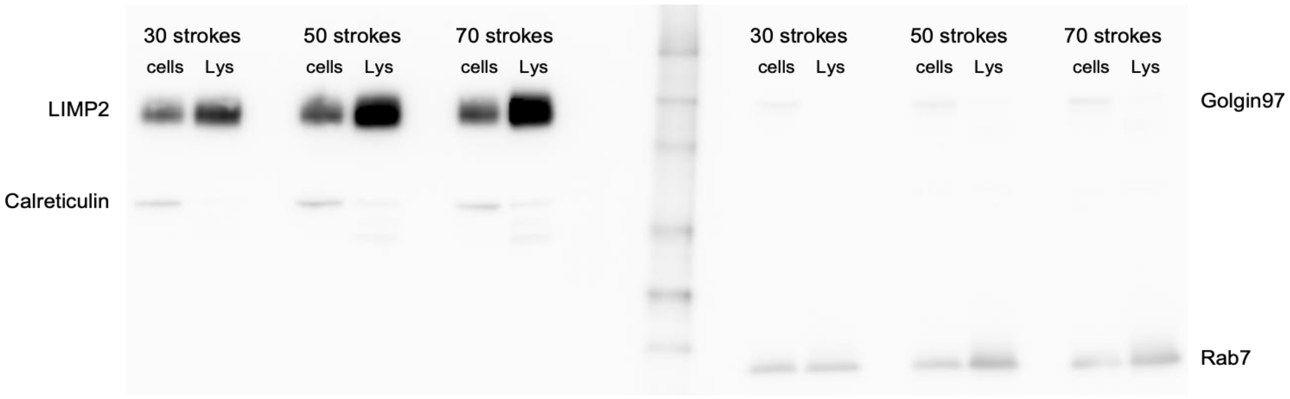

(b)
