# Supplementary material for: Imipramine Treatment Alters Sphingomyelin, Cholesterol, and Glycerophospholipid Metabolism in Isolated Macrophage Lysosomes
Source: Biomolecules. 2023 Dec 1;13(12):1732. doi: 10.3390/biom13121732 (PMC10742328; doi:10.3390/biom13121732)

Figure S2. Photograph illustrating similarity in band sedimentation between lysosomes isolated from control and imipramine-treated mexAM.

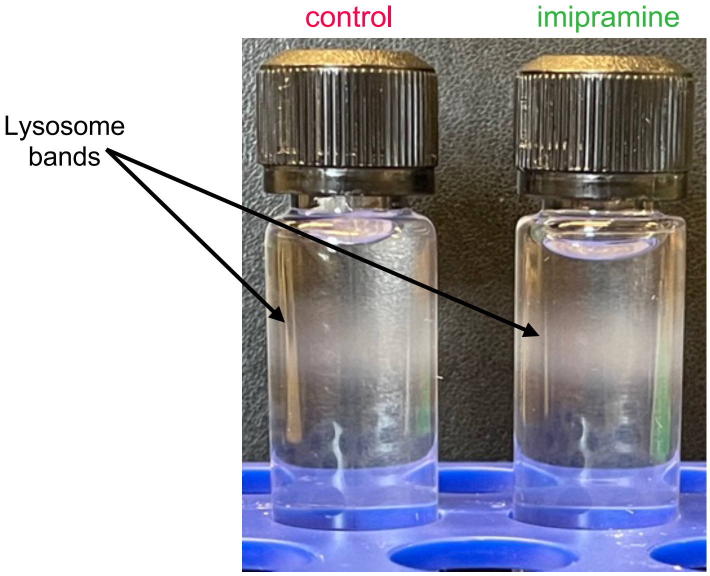

Supplement: Supplementary file 1 [file biomolecules-13-01732-s001.zip › Figure S2.pdf]
