# Supplementary material for: Imipramine Treatment Alters Sphingomyelin, Cholesterol, and Glycerophospholipid Metabolism in Isolated Macrophage Lysosomes
Source: Biomolecules. 2023 Dec 1;13(12):1732. doi: 10.3390/biom13121732 (PMC10742328; doi:10.3390/biom13121732)

Figure S3. Original western blot image (a) and brightness adjusted image (b) corresponding to Figure 1. The brightness/contrast maximum was adjusted from 65535 to 38943 for the entire image using ImageJ to ensure complete protein band detection and to remove pixelation in LIMP2 protein bands. Two PVDF membranes were imaged simultaneously under the same conditions to produce one image. Lysosomes were isolated from mexAM using density gradient ultracentrifugation. Equal amounts of protein (10 μg) were loaded into each lane. Bands represent organelle specific antibodies: lysosomes (LIMP2), ER (Calreticulin), Golgi, (Golgin97), and late Endosomes (Rab7). Cell lysates (Cells), lysosomes from control cells (C), lysosomes from imipramine-treated cells (IMP).

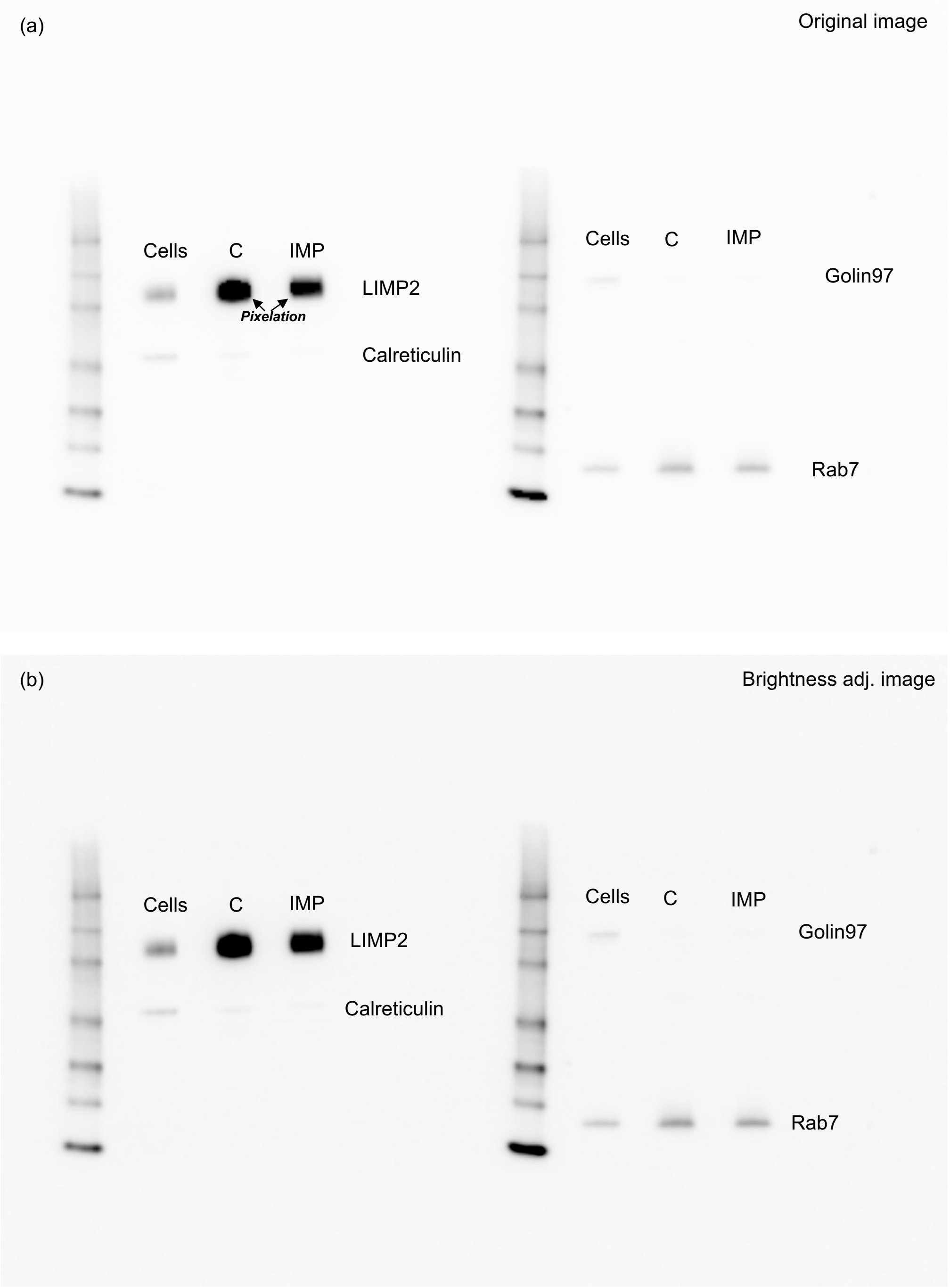

Supplement: Supplementary file 1 [file biomolecules-13-01732-s001.zip › Figure S3.pdf]
