# Supplementary material for: Imipramine Treatment Alters Sphingomyelin, Cholesterol, and Glycerophospholipid Metabolism in Isolated Macrophage Lysosomes
Source: Biomolecules. 2023 Dec 1;13(12):1732. doi: 10.3390/biom13121732 (PMC10742328; doi:10.3390/biom13121732)

Supplemental Figure S4. Representative VV and VH fits for time-resolved anisotropy measurements of Di-4ANEPPDHQ in lysosomes.

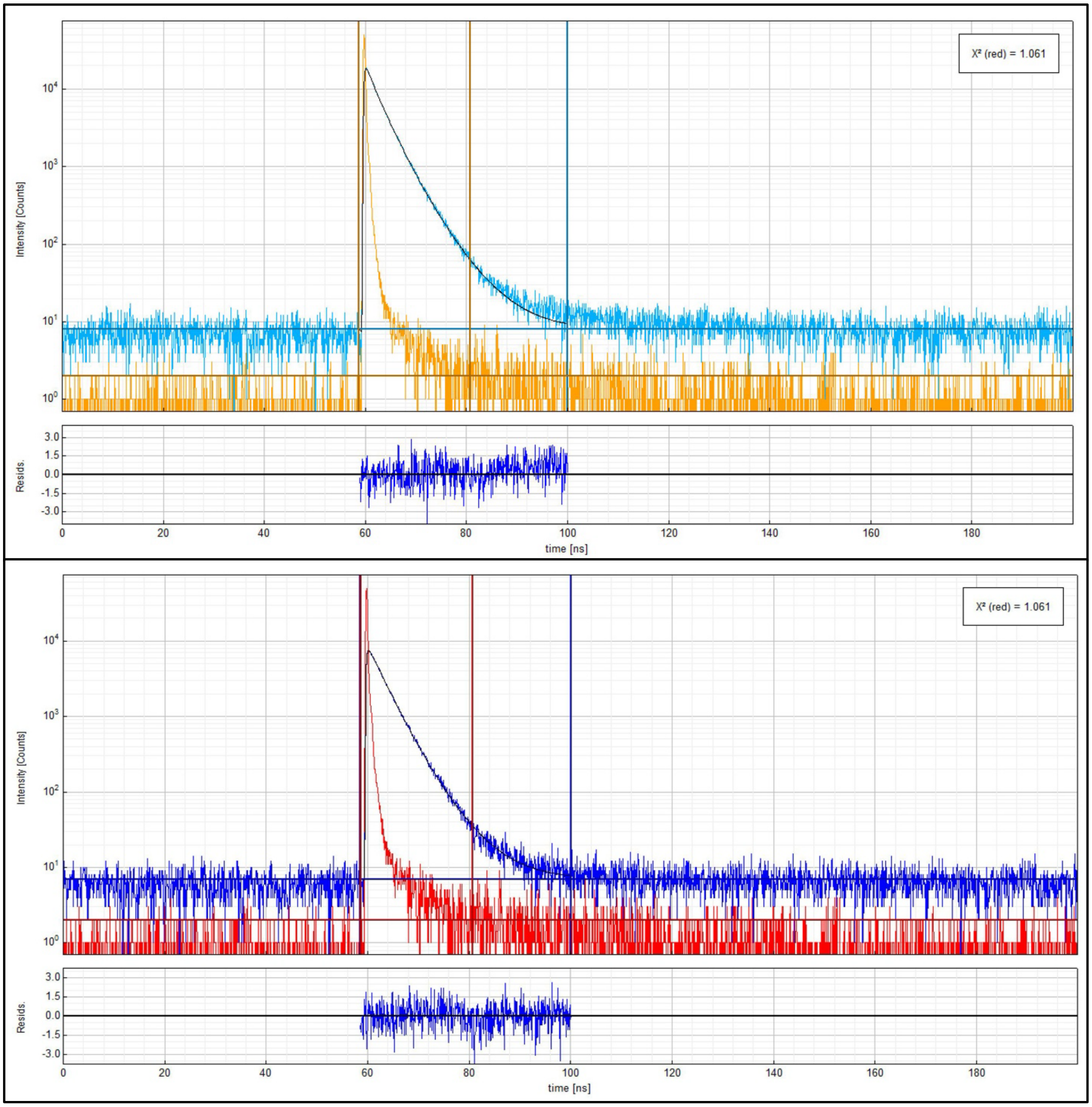

Supplement: Supplementary file 1 [file biomolecules-13-01732-s001.zip › Figure S4.pdf]
