# Supplementary material for: Imipramine Treatment Alters Sphingomyelin, Cholesterol, and Glycerophospholipid Metabolism in Isolated Macrophage Lysosomes
Source: Biomolecules. 2023 Dec 1;13(12):1732. doi: 10.3390/biom13121732 (PMC10742328; doi:10.3390/biom13121732)
Supplement: Supplementary file 1 [file biomolecules-13-01732-s001.zip › Table S1.pdf]

Table S1. Quantitation of Dounce strokes based on mean gray values of western blot protein bands. Only control lysosomes (C. Lysosomes) were analyzed for optimal Dounce stroke determination.

			(255 - mean gray value)		(255 – Background)	(Inv. gray value – Inv. Background)
Cell lysate	# of Dounce strokes	Mean gray value	Inverted gray value	Background	Inverted Background	Net gray value
LIMP2	30	222.803	32.197	247.986	7.014	25.183
	50	204.347	50.653	247.997	7.003	43.65
	70	205.359	49.641	247.957	7.043	42.598
Calreticulin (Cal.)	30	247.425	7.575	248	7	0.575
	50	247.114	7.886	248	7	0.886
	70	247.407	7.593	248	7	0.593
C. Lysosomes						
LIMP2	30	190.433	64.567	248	7	57.567
	50	135.735	119.265	247.949	7.051	112.214
	70	135.098	119.902	248	7	112.902
Calreticulin (Cal.)	30	248	7	248	7	0.067
	50	247.93	7.07	247.943	7.057	0.12
	70	247.906	7.094	248	7	0.094
		Cell lysate: Net LIMP2/Net Cal.	C. Lysosomes: Net LIMP2/Net Cal.	Cell lysate: (Net LIMP2/Net Cal.) / Lysosomes: (Net LIMP2/Net Cal.)	**Approx. Fold-purity**	
	30	43.80	859.21	19.62	**~20**	
	50	49.27	935.12	18.98	**~19**	
	70	71.83	1201.09	16.72	**~17**	
