# Supplementary material for: Imipramine Treatment Alters Sphingomyelin, Cholesterol, and Glycerophospholipid Metabolism in Isolated Macrophage Lysosomes
Source: Biomolecules. 2023 Dec 1;13(12):1732. doi: 10.3390/biom13121732 (PMC10742328; doi:10.3390/biom13121732)
Supplement: Supplementary file 1 [file biomolecules-13-01732-s001.zip › Table S2.pdf]

Table S1. Quantitation of western blot image corresponding to Figure 1. Lysosomes from control cells (C. Lysosomes), lysosomes from imipramine-treated cells (IMP. Lysosomes).

			(255 - mean gray value)		(255 – Background)	(Inv. gray value – Inv. Background)
	Sample	Mean gray value	Inverted gray value	Background	Inverted Background	Net gray value
LIMP2	Cell lysates	213.160	41.840	245.967	9.033	32.807
	C. Lysosomes	83.586	171.414	244.140	10.860	160.554
	IMP. Lysosomes	113.284	141.716	245.149	9.851	131.865
Calreticulin (Cal.)	Cell lysates	241.141	13.859	246.319	8.681	5.178
	C. Lysosomes	244.568	10.432	246.037	8.963	1.469
	IMP. Lysosomes	245.535	9.465	246.632	8.368	1.097
		Net LIMP2/Net Cal.	(Lysosomes: LIMP2/Cal.) / (Cells: LIMP2/Cal.)	**Approx. Fold-purity**		
	Cell lysates	6.34				
	C. Lysosomes	109.29	17.25	**~17**		
	IMP. Lysosomes	120.21	18.97	**~19**		
