# Supplementary material for: Imipramine Treatment Alters Sphingomyelin, Cholesterol, and Glycerophospholipid Metabolism in Isolated Macrophage Lysosomes
Source: Biomolecules. 2023 Dec 1;13(12):1732. doi: 10.3390/biom13121732 (PMC10742328; doi:10.3390/biom13121732)
Supplement: Supplementary file 1 [file biomolecules-13-01732-s001.zip › Table S3.pdf]

Table S3. Lipid species fold-change data corresponding to Figure 4.

Lipid name	MRM	Control 1 (fold-change)	Control 2 (fold-change)	Control 3 (fold-change)	IMP 1 (fold-change)	IMP 2 (fold change)	IMP 3 (fold-change)	IMP/Control (avg fold-change)	p-value
CE(18:1)	668.634574 -> 369.2	0.72006799	1.15664077	1.12329124	2.71221034	2.72566066	2.98880708	2.80889269	0.00040935
CE(20:4)	690.618874 -> 369.2	0.77434639	1.07740284	1.14825077	2.15012617	2.34216913	2.21704916	2.23644815	0.00063734
CE(18:2)	666.618874 -> 369.2	0.76171344	1.15160138	1.08668518	1.7739422	1.93378373	2.11635099	1.94135897	0.00380066
PC(33:3),PC(O-34:3),PC(P-34:2)	742.575025 -> 184.1	1.01311388	1.06397692	0.92290921	1.84938302	1.73745144	1.58629201	1.72437549	0.00112065
PC(31:2),PC(O-32:2),PC(P-32:1)	716.559425 -> 184.1	0.94551719	0.99209236	1.06239045	1.78149503	1.55679154	1.69813691	1.67880783	0.00077844
SM(d18:1/19:0)	745.622325 -> 184.1	0.97905981	0.99792749	1.0230127	1.52380743	1.53370858	1.5688503	1.5421221	8.3862E-06
PC(35:5),PC(O-36:5),PC(P-36:4)	766.575025 -> 184.1	1.0927749	0.98152657	0.92569853	1.5481501	1.43460004	1.54005701	1.50760238	0.00115601
PC(40:5)	836.616925 -> 184.1	1.03919479	0.95205393	1.00875128	1.50517059	1.53752504	1.47223217	1.50497593	9.1193E-05
PC(33:2),PC(O-34:2),PC(P-34:1)	744.590725 -> 184.1	0.96347958	1.02482657	1.01169385	1.48708535	1.45972543	1.45429226	1.46703435	2.5277E-05
SM(d16:1/18:1)	701.559725 -> 184.1	0.9790005	1.04374436	0.97725514	1.34032785	1.53302683	1.4289015	1.43408539	0.00191631
PC(38:4)	810.601325 -> 184.1	1.15639787	0.91701605	0.92658608	1.43304601	1.45636216	1.35484984	1.41475267	0.00784838
PC(38:5)	808.585625 -> 184.1	0.96101301	1.01804495	1.02094205	1.35463362	1.45814663	1.35002193	1.38760073	0.00065537
PC(38:3)	812.616925 -> 184.1	0.8777381	1.08497361	1.0372883	1.35182041	1.4952903	1.25497156	1.36736076	0.01730358
PC(40:6)	834.601325 -> 184.1	0.99097621	1.04225871	0.96676508	1.2336393	1.37495299	1.30772594	1.30543941	0.00277591
PC(36:3),PC(P-37:2)	784.622025 -> 184.1	0.9618184	0.99610219	1.04207941	1.24678674	1.23525737	1.37904695	1.28703035	0.00513517
PC(37:3),PC(O-38:3),PC(P-38:2)	798.637625 -> 184.1	1.07035731	0.96499101	0.96465168	1.20918116	1.36710374	1.27481124	1.28369871	0.00797417
PC(35:3),PC(O-36:3),PC(P-36:2)	770.606325 -> 184.1	0.98825185	0.98302325	1.02872489	1.28231705	1.25596376	1.2489161	1.26239897	0.00011947
SM(d18:1/17:0)	717.591025 -> 184.1	1.06006485	1.01452247	0.92541268	1.19006777	1.16877848	1.38063342	1.24649322	0.03431338
SM(d16:0/23:0)	775.669325 -> 184.1	0.89405075	1.02032331	1.08562594	1.24823821	1.19377925	1.21113411	1.21771719	0.02041981
SM(d16:1/20:1)	729.591025 -> 184.1	0.94374308	1.10619831	0.95005862	1.29064854	1.19185582	1.1695543	1.21735289	0.02854508
PC(39:5),PC(O-40:5),PC(P-40:4)	822.637625 -> 184.1	0.99934955	0.95771943	1.04293102	1.22206393	1.23693829	1.12760117	1.19553446	0.00974822
SM(d16:1/22:1)	757.622325 -> 184.1	0.93805479	0.98515717	1.07678804	1.12716621	1.12284009	1.2141384	1.1547149	0.03737217

SM(d18:2/22:1)	783.638025 -> 184.1	0.97054981	0.99509787	1.03435233	1.08853952	1.16591325	1.20889373	1.15444883	0.01785316
PC(39:4),PC(O-40:4),PC(P-40:3)	824.653325 -> 184.1	0.99786194	1.04624947	0.95588859	1.14735737	1.10662931	1.18484186	1.14627618	0.01329072
PC(36:4),PC(O-37:4)	782.606325 -> 184.1	0.99371926	1.01659547	0.98968527	1.07453733	1.20627942	1.1305844	1.13713372	0.02469352
PC(35:4),PC(O-36:4),PC(P-36:3)	768.590725 -> 184.1	1.01506611	0.98523927	0.99969462	1.14255071	1.11647427	1.14623759	1.13508752	0.00044561
LPC(18:1),PC(O-18:1),PC(P-18:0)	522.355925 -> 184.1	1.02641769	1.0087661	0.96481621	1.15471653	1.16540127	1.07923872	1.13311884	0.01524558
PC(36:8),PC(35:1),PC(O-36:1),PC(P-36:0)	774.637625 -> 184.1	0.96617632	0.99863124	1.03519244	1.07950843	1.12333551	1.15670198	1.11984864	0.01610865
PC(40:7),PC(39:0),PC(O-40:0)	832.715925 -> 184.1	0.96773604	1.01946457	1.01279939	1.05260714	1.15558863	1.11405409	1.10741662	0.03432882
SM(d17:1/26:1)	827.700625 -> 184.1	0.9965701	1.05095055	0.95247934	1.10469022	1.08207461	1.12972039	1.10549507	0.02895684
PC(38:7),PC(37:0),PC(O-38:0)	804.684625 -> 184.1	0.96292051	1.01246327	1.02461622	0.87146599	0.93050173	0.86480013	0.88892262	0.01685875
SM(d16:1/18:0)	703.575425 -> 184.1	1.072063	0.95290035	0.97503666	0.89294773	0.86503548	0.87366769	0.87721697	0.03069839
PC(32:1),PC(O-33:1),PC(P-33:0)	732.590725 -> 184.1	1.02698981	0.99835906	0.97465113	0.89954686	0.86795088	0.85940049	0.87563274	0.00306691
SM(d18:0/17:0)	719.606725 -> 184.1	0.94526359	1.02406246	1.03067396	0.90693754	0.79497577	0.89299364	0.86496898	0.03900167
PC(40:2)	842.663925 -> 184.1	1.02335945	1.05789014	0.91875041	0.82099378	0.89022306	0.83424103	0.84848596	0.03196359
PC(32:0),PC(O-33:0)	734.606325 -> 184.1	1.06930781	0.97179357	0.95889862	0.80732079	0.84800849	0.85166079	0.83566336	0.01200429
SM(d18:0/26:1(17Z))	843.731925 -> 184.1	0.89430598	1.03022789	1.07546612	0.87478042	0.76308198	0.76019723	0.79935321	0.03879712
1-O-carboceroyl-Cer(d18:1/18:0)	972.968725 -> 264.4	1.1235224	1.04850991	0.82796769	0.75389217	0.63452298	0.76909428	0.71916981	0.04618034
Cer(d18:1/18:0)	566.551225 -> 264.4	1.06371752	1.00015228	0.9361302	0.8013944	0.66963957	0.67323861	0.71475753	0.0074075
SM(d18:2/18:1)	727.575425 -> 184.1	1.19112163	0.94436633	0.86451204	0.75048893	0.72269236	0.6161329	0.69643806	0.04636557
PC(26:0)	650.476125 -> 184.1	1.19359096	0.96810097	0.83830807	0.6091425	0.7627107	0.64676864	0.67287395	0.04504951
LPC(16:0),PC(O-16:0),LPC(O-17:0)	496.376725 -> 184.1	1.02653109	1.03697935	0.93648956	0.6550728	0.67400579	0.68119416	0.67009092	0.00055183
CE(19:0)	684.665874 -> 369.2	1.26992827	0.86259984	0.8674719	0.50428173	0.56414667	0.48931527	0.51924789	0.02463047
