# Supplementary material for: Imipramine Treatment Alters Sphingomyelin, Cholesterol, and Glycerophospholipid Metabolism in Isolated Macrophage Lysosomes
Source: Biomolecules. 2023 Dec 1;13(12):1732. doi: 10.3390/biom13121732 (PMC10742328; doi:10.3390/biom13121732)
Supplement: Supplementary file 1 [file biomolecules-13-01732-s001.zip › Table S4.pdf]

Table S4. Absolute intensities of lipid species corresponding to Figure 4.

Lipid name	MRM	Control 1 (Absolute intensity)	Control 2 (Absolute intensity)	Control 3 (Absolute intensity)	IMP 1 (Absolute intensity)	IMP 2 (Absolute intensity)	IMP 3 (Absolute intensity)
**CE(18:1)**	668.634574 -> 369.2	6830.860451	7157.360493	8327.700630	7831.400547	33301.442230	41471.562780
**CE(18:2)**	666.618874 -> 369.2	6653.840473	6703.180458	7340.720520	6707.500477	19283.641320	26049.321860
**CE(19:0)**	684.665874 -> 369.2	9171.520645	14913.481060	7337.640518	7145.340511	7315.320534	10141.240730
**CE(20:4)**	690.618874 -> 369.2	6063.560429	6773.480488	6826.560482	7045.000488	23232.761680	31361.302160
**SM(d16:1/18:1)**	701.559725 -> 184.1	1464.580097	10247.22080	12521.940930	14734.901210	21823.301440	29129.082440
**SM(d16:1/18:0)**	703.575425 -> 184.1	4867.000328	1371124.438000	1396877.680000	1796360.898000	1776514.799000	2008372.914000
**SM(d18:1/17:0)**	717.591025 -> 184.1	1289.940083	13480.881040	14787.741180	16952.641140	23542.022100	26981.862060
**SM(d18:0/17:0)**	719.606725 -> 184.1	1307.780090	42615.062880	52916.483590	66934.124910	63602.444900	65060.585010
**SM(d18:2/18:1)**	727.575425 -> 184.1	1214.260086	1489.120098	1353.220089	1556.900101	1459.500107	1640.140102
**SM(d16:1/20:1)**	729.591025 -> 184.1	1344.200089	5367.420334	7211.060627	7783.560635	11418.420960	12305.221070
**SM(d18:1/19:0)**	745.622325 -> 184.1	1213.460083	32904.043000	38440.882690	49526.443980	79662.944160	93570.026570
**SM(d16:1/22:1)**	757.622325 -> 184.1	1193.720085	7785.720524	9371.960564	12874.100980	14552.740790	16917.741370
**SM(d16:0/23:0)**	775.669325 -> 184.1	1291.740086	11350.780900	14847.580940	19854.541450	24651.722290	27513.222450
**SM(d18:2/22:1)**	783.638025 -> 184.1	1542.780113	20505.401600	24097.421540	31480.022240	35775.10213	44716.783700
**SM(d17:1/26:1)**	827.700625 -> 184.1	1298.220081	4306.360298	5205.220360	5928.900452	7425.560570	8488.160549
**SM(d18:0/26:1(17Z))**	843.731925 -> 184.1	1278.740089	4478.760372	5913.7004810	7758.620518	6814.860565	6937.400383
**Cer(d18:1/18:0)**	566.551225 -> 264.4	1198.560078	1212.500088	1306.700096	1537.120110	1420.980110	1385.640095
**1-O-carboceroyl-Cer(d18:1/18:0)**	972.968725 -> 264.4	1207.700096	1216.220081	1300.940102	1291.100086	1269.480087	1246.900089
**LPC(16:0),PC(O-16:0),LPC(O-17:0)**	496.376725 -> 184.1	5063.100388	33468.762810	53341.825150	66805.843880	43126.262580	56487.54402
**LPC(18:1),PC(O-18:1),PC(P-18:0)**	522.355925 -> 184.1	4538.940273	18710.381240	29012.101960	38481.062870	42502.903020	54607.904000
**PC(26:0)**	650.476125 -> 184.1	2299.460144	6867.500481	8788.080772	10553.360780	7076.960648	11280.400760
**PC(31:2),PC(O-32:2),PC(P-32:1)**	716.559425 -> 184.1	3153.320232	10372.120650	17170.401250	25499.181960	39460.942100	43898.542830
**PC(32:1),PC(O-33:1),PC(P-33:0)**	732.590725 -> 184.1	74442.22596	534230.307600	819368.686000	1109316.675000	944867.449400	1160590.597000
**PC(32:0),PC(O-33:0)**	734.606325 -> 184.1	21482.94151	93741.967210	134411.229800	183928.053900	142909.850600	191096.634000
**PC(33:3),PC(O-34:3),PC(P-34:2)**	742.575025 -> 184.1	1418.820099	6739.440453	11166.780790	13432.860800	24841.461690	29709.802400
**PC(33:2),PC(O-34:2),PC(P-34:1)**	744.590725 -> 184.1	9586.580612	71789.664540	120475.849300	164934.870300	223738.133500	279583.861100
**PC(35:5),PC(O-36:5),PC(P-36:4)**	766.575025 -> 184.1	2361.340153	10795.020800	15297.661420	20008.121140	30880.942320	36428.821700
**PC(35:4),PC(O-36:4),PC(P-36:3)**	768.590725 -> 184.1	3152.400230	16303.681110	24966.822070	35131.982440	37055.402280	46095.822910

**PC(35:3),PC(O-36:3),PC(P-36:2)**	770.606325 -> 184.1	3424.260216	18438.121360	28936.302270	41994.483220	48309.123030	60234.824780
**PC(36:8),PC(35:1),PC(O-36:1),PC(P-36:0)**	774.637625 -> 184.1	5811.780411	30028.681560	48968.323060	70395.504750	67747.085780	89745.126740
**PC(36:4),PC(O-37:4)**	782.606325 -> 184.1	8782.440590	42878.704040	69208.003910	93437.026790	93623.345550	133797.308200
**PC(36:3),PC(P-37:2)**	784.622025 -> 184.1	8593.440643	67960.885370	111045.267400	161105.633200	177886.433900	224359.733400
**PC(37:3),PC(O-38:3),PC(P-38:2)**	798.637625 -> 184.1	1657.880127	4454.680305	6336.380356	8784.200638	10161.640790	14625.500760
**PC(38:7),PC(37:0),PC(O-38:0)**	804.684625 -> 184.1	2047.580128	5886.760441	9765.520863	13705.381070	10757.740720	14622.600780
**PC(38:5)**	808.585625 -> 184.1	6339.420425	30925.662440	51687.664270	71884.423890	88023.027180	120618.092300
**PC(38:4)**	810.601325 -> 184.1	7284.400452	26153.682170	32721.462400	45851.684040	65444.084560	84667.445720
**PC(38:3)**	812.616925 -> 184.1	3803.660301	14359.860980	28004.941600	37130.322920	44656.943100	62882.864490
**PC(39:5),PC(O-40:5),PC(P-40:4)**	822.637625 -> 184.1	1699.100124	5436.120350	8219.400612	12412.840810	13423.021100	17295.821150
**PC(39:4),PC(O-40:4),PC(P-40:3)**	824.653325 -> 184.1	1284.360085	2529.460178	4184.300335	5301.620354	5872.740372	7210.760460
**PC(40:7),PC(39:0),PC(O-40:0)**	832.715925 -> 184.1	2701.080189	12581.581050	20911.281720	28810.161680	27633.081830	38619.182510
**PC(40:6)**	834.601325 -> 184.1	2870.380184	9446.420692	15675.081000	20163.641510	23745.261380	33690.962210
**PC(40:5)**	836.616925 -> 184.1	1498.940102	5998.440441	8670.280579	12740.000700	17543.321430	22813.141870
**PC(40:2)**	842.663925 -> 184.1	1401.180115	3892.340233	6348.240482	7645.820541	6305.320496	8703.680588
